# Straightforward selection of broadly neutralizing single-domain antibodies targeting the conserved CD4 and co-receptor binding sites of HIV-1 gp120

**DOI:** 10.1186/1742-4690-9-S2-P214

**Published:** 2012-09-13

**Authors:** J Matz, P Kessler, J Bouchet, O Combes, D Baty, L Martin, S Benichou, P Chames

**Affiliations:** 1 INSERM, Marseille, France

## Background

The interaction of gp120 with CD4 is the first step of HIV cycle. It is the pivotal event allowing the entry of HIV into CD4+ cells. gp120 is the main target for neutralizing antibodies (nAb) but most of its accessible epitopes are highly variable and thus, HIV can bypass these nAbs.

Single domain antibodies (sdAb) derived from llama antibodies bind their antigen without requiring variable domains pairing. They are able to bind unconventional epitopes, like those present in protein cavities. Their small size (13 kDa) allows them to accede to very narrow space, such as the space between virus and cell membranes during infection. SdAbs are highly stable, easy to clone and produce in large amounts.

## Methods

Using trimeric gp140, free or bound to a CD4 mimic, as immunogens in llamas, we selected a panel of broadly neutralizing single-domain antibodies that bind either to the CD4 or to the co-receptor binding sites (CD4BS and CoRBS, respectively).

## Results

When analyzed as monomers or as homo- or hetero-multimers, the best sdAb candidates could not only neutralize viruses carrying subtype B envelopes, corresponding to the Env molecule used for immunization and selection, but were also efficient in neutralizing a broad panel of envelopes from subtypes A, C, G and CRF01_AE. Interestingly, sdAb multimers exhibited a broader spectrum of neutralizing activity than the parental sdAb monomers.

## Conclusion

The extreme stability and high recombinant production yield combined to their broad neutralization capacity make these sdAbs new potential microbicide candidates for HIV-1 transmission prevention.

